# Morphological and Molecular Analysis of the *Nematostella vectensis* Cnidom

**DOI:** 10.1371/journal.pone.0022725

**Published:** 2011-07-28

**Authors:** Claudia Zenkert, Toshio Takahashi, Mark-Oliver Diesner, Suat Özbek

**Affiliations:** 1 Department for Molecular Evolution and Genomics, Centre for Organismal Studies, University of Heidelberg, Heidelberg, Germany; 2 Suntory Foundation for Life Sciences, Bioorganic Research Institute, Osaka, Japan; University of Otago, New Zealand

## Abstract

The starlet sea anemone *Nematostella vectensis* is an emerging model organism for developmental and evolutionary biology. Due to the availability of genome data and its amenability to genetic manipulation *Nematostella* serves as a source for comparative molecular and phylogenetic studies. Despite this fact, the characterization of the nematocyst inventory and of nematocyst-specific genes is still fragmentary and sometimes misleading in this cnidarian species. Here, we present a thorough qualitative and quantitative analysis of nematocysts in *Nematostella vectensis*. In addition, we have cloned major nematocyst components, *Nematostella* minicollagens 1, 3 and 4, and show their expression patterns by in situ hybridization and immunocytochemistry using specific antibodies. Our data provides tools and insights for further studies on nematocyst morphogenesis in *Nematostella* and comparative evolution in cnidarians.

## Introduction

Due to their phylogenetic position as a sister group to bilatarians cnidarians have been of interest for the exploration of animal body patterning and embryonic development [1]. The genomes of *Hydra* [2] and *Nematostella* [3] have been sequenced and provide a basis for extensive comparative studies. The establishment of transgenesis in both species has opened additional experimental possibilities for functional genetic approaches [4], [5]. Despite this progress there is only limited information on nematocysts in *Nematostella* compared to the extensive studies performed in *Hydra* [6]. Nematocysts are the defining predatory organelles of the phylum cnidaria and represent a complex secretory product of the Golgi apparatus [7]. They are composed of a cylindrical capsule body that elongates into a long tubule, which is coiled up in the capsule matrix. This basic structure varies between different cnidarian species, but there is a general tendency towards higher complexity in *medusozoa* compared to *anthozoa* [8]. Nematocyst production involves the coordinated secretion of proteins into the growing nematocyst vesicle, where these are assembled to form the collagenous capsule structure. Mature nematocysts are connected to a mechanosensory cnidocil apparatus that triggers their ultra-fast discharge process [9].

In *Hydra*, four types of nematocysts have been described and several molecular capsule components have been extensively characterized. In particular, the large minicollagen family, which represents the major structural constituent of the organelle, has been the subject of numerous biochemical and structural studies [10], [11], [12], [13].

Here, we present the first extensive description of the *Nematostella* cnidom and characterize the expression patterns and localization of several isolated minicollagens. Our work provides evidence for a basal nematocyst structure in the anthozoan-specific spirocysts and tools for further comparative studies on nematocyst development and evolution within the cnidarian clade.

## Results

### Morphological characterization of nematocyst types in *Nematostella*

**Figure 1:**
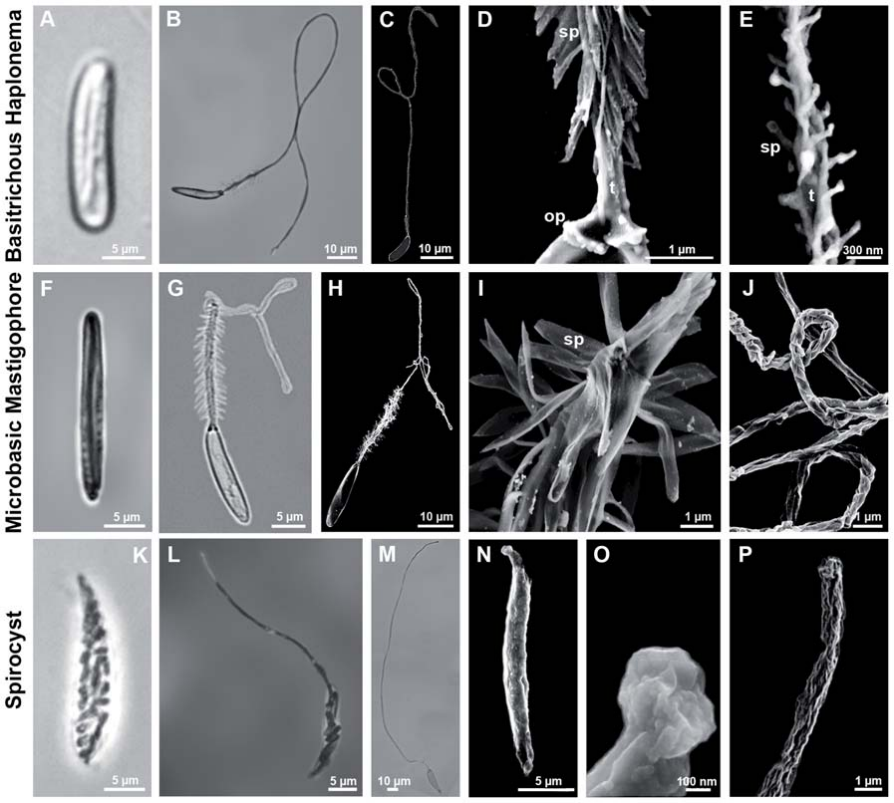
Capsule types in *Nematostella vectensis*. A–E. Basitrichous haplonemas. F–J. Microbasic mastigophores. K–P. Spirocysts. A, B. Light microscopic images of an undischarged (A) and a fully discharged (B) basitrichous haplonema. C–E. Scanning electron microscopic (SEM) images of haplonemas. C. Fully discharged haplonema. D. Detail of C, showing the basal spines (sp) and the opercular apparatus (op). E. Detail of the distal part of the tubule (t), revealing reduced spines (sp). F–J. Microbasic mastigophores. F, G. Undischarged (F) and fully discharged (G) capsule. H–J. SEM images of microbasic mastigophores. H. Fully discharged capsule. I. Detail of H showing the flexible spines (sp). J. Detail of the triangular tubule showing both the coiled and the distal uncoiled structure. K–P. Spirocysts. K–M. Light microscopic images of an undischarged (K), partly (L) and fully discharged spirocyst (M). N–P. SEM images of spirocysts. N. Undischarged spirocyst. O. Detail showing the closed opening of the capsule body. P. Distal end of discharged tubule. Scale bars are 10 µm (B, C, H, M), 5 µm (A, F, G, K, L, N), 1 µm (D, I, J, P), 300 nm (E), and 100 nm (O).

Nematocysts of *Nematostella vectensis* were isolated by Percoll gradient centrifugation and characterized morphologically using light and scanning electron microscopy (SEM). We were able to distinguish three different capsule types according to the classification of Weill [14], [15] (Figure 1): (i) the smallest and most common nematocyst type is the basitrichous haplonema (Figure 1A–E). It is about 12 µm long and 2 µm in width. The tubule has a diameter of 0.5 µm, with a total length of 90–110 µm (Figure 1B, C). Dense spines of 1–2 µm length are arranged in spirals along a stretch of 20–25 µm at the base of the tubule (Figure 1B–D). SEM analysis revealed smaller spines (∼0.1 µm) (Figure 1E) covering up to 2/3 of the total tubule length; (ii) the larger microbasic mastigophores (Figure 1F–J) are 17–22 µm long and 3 µm in width. The clearly discernible tubule base (30–40 µm) is about 1.5 µm in diameter and covered with dense spirals of spines of 1–4 µm length (Figure G–I). The tubule has a total length of about 180 µm with a narrower and smooth distal part (Figure 1H, J); (iii) spirocysts are the characteristic cone-shaped nematocysts of anthozoans. In *Nematostella* they are about 25–30 µm long and thus represent the largest capsule type (Figure 1K–P). The spineless tubule (Figure 1P) is visible in typical large coils inside the capsule body and has an average diameter of 1 µm and a length of about 180 µm (Figure 1K–M). Spirocysts are distinctive in exhibiting a less dense capsule wall than other nematocyst types. This apparent fragility is also reflected by the fact that most spirocysts are disrupted by the isolation procedure and quantification has to rely on a careful maceration procedure. SEM analysis revealed that spirocysts lack an opercular structure formed by the capsule wall (Figure 1N, O). Rather, capsule closure appears to be realized by folds at the base of the inverted tubule (Figure 1O) suggesting a continuous structure between tubule and capsule body for this nematocyst type.

### Quantitative analysis of nematocysts at different developmental stages

**Figure 2:**
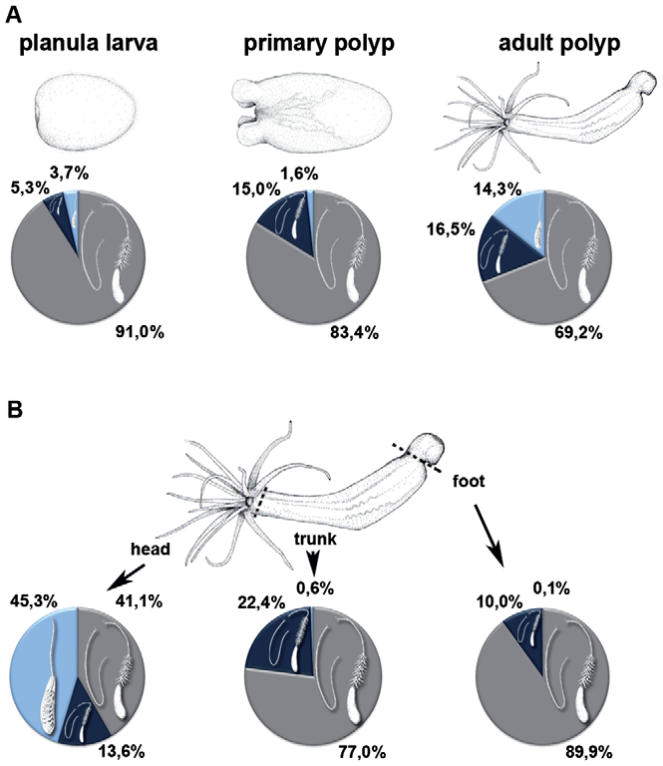
Quantitative distribution of nematocysts in *Nematostella* polyps. A. Distribution of nematocyst types in different developmental stages. B. Distribution of nematocyst types in different body parts of Nematostella vectensis.

To analyze whether the composition of nematocyst types changed during development we quantified nematocysts at different stages of *Nematostella* morphogenesis (Figure 2A, Table S1). In planula larvae basitrichous haplonemas clearly dominated, constituting 91% of all capsule types, while mastigophores and spirocysts were represented only to 5.3% and 3.7%, respectively. The percentage of basitrichous haplonemas was slightly reduced in primary (83.4%) and adult polyps (69.2%), whereas microbasic mastigophores were almost constant at 15% and 16.5%, respectively. Spirocysts stayed at a low level (1.6%) in primary polyps but were increased to 14.3% in adult animals. Considering the total tissue of the animal, nematocytes made up 4–5% of all cells at each developmental stage.

To analyze the distribution of nematocyst types along the different body parts of the animal we quantified capsules in head, body and foot regions of adult polyps (Figure 2B). Although mature nematocysts in *Nematostella* are distributed all along the body axis, the highest density of capsules is found in the tentacles, the tentacle base and the hypostomal area. In the head region, the majority of nematocysts (45.3%) are spirocysts, followed by basitrichous haplonemas (41.1%) and microbasic mastigophores (13.6%). Nematocysts in the body column and foot region are mainly basitrichous haplonemas (77% and 89.9%) and microbasic mastigophores (22.4% and 9.9%), suggesting that spirocysts and microbasic nematocysts are offensive capsule types used for prey capture, while basitrichous haplonemas mainly have a defensive function against predators.

### Isolation of *Nematostella* minicollagen genes

Molecules of the minicollagen family are major structural constituents of nematocysts and have been identified in all cnidarian genomes analyzed so far [8]. Minicollagens are composed of a central collagen triple helix flanked by polyproline stretches and short cysteine-rich domains (CRDs) with a conserved cysteine pattern (CX_3_CX_3_CX_3_CX_3_CC) [12]. In *Hydra*, 17 members of this protein family have been isolated while in *Nematostella* only 5 could be identified in the genome database, reflecting the lower complexity of anthozoan nematocysts [8]. As minicollagens are important markers of nematocyst morphogenesis we isolated the *Nematostella* minicollagen genes by cloning from cDNA and raised peptide antibodies against their CRD peptides, which serve as signature domains. We succeeded in cloning minicollagens 1, 3, and 4 (NvNCol-1; NvNCol-3; NvNCol-4) while the predicted minicollagen gene sequences of NvNCol-5 and NvNCol-6 could not be amplified. This is probably due to highly homologous sequences between minicollagens and a lower abundance of the respective cDNAs.

**Figure 3:**
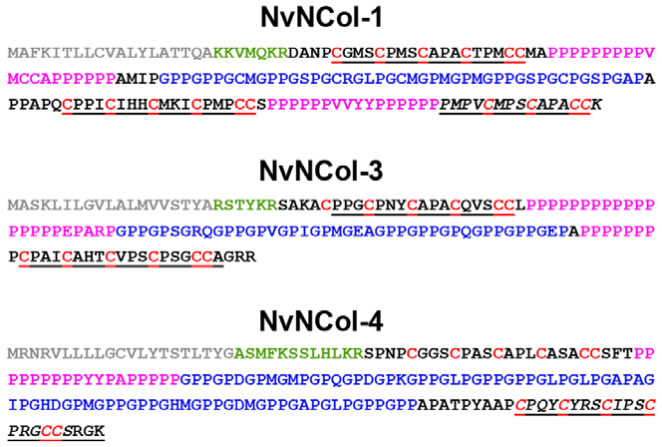
Amino acid sequences of *Nematostella* minicollagens. Protein sequences of minicollagens NvNCol-1, NvNCol-3, and NvNCol-4. Signal peptides in grey; propeptide in green; poly-proline stretches in pink; cysteine-rich domains (CRDs) are indicated in underlines, cysteine residues are in red. The C terminal CRDs printed in italics were chosen for raising the polyclonal antibodies.

The full-length minicollagen sequences are shown in Figure 3. NvNCol-1 has an open reading frame (ORF) of 516 nucleotides, coding for 172 amino acids. NvNCol-3 is slightly shorter with 429 nucleotides (143 amino acids) and NvNCol-4 comprises 570 nucleotides coding for 190 amino acids. All three identified NvNCols have individual signal peptides with 19–21 amino acids followed by a propeptide sequence terminating with the characteristic lysine-arginine (KR) cleavage site. Their domain composition includes a central collagen sequence comprising between 14–27 Gly-X-Y repeats flanked by variable polyproline stretches and terminal CRDs. NvNCol-3 and NvNCol-4 possess single canonical CRDs while NvNCol-1 shows a CRD duplication at the C-terminus with the second CRD lacking the first two cysteines of the conserved pattern as in *Hydra* NCol-15 [13]. In contrast to *Hydra*, the minicollagen genes in *Nematostella* are positioned on different scaffolds and do not occur in clusters of tandemly repeated genes sharing a common signal peptide [8].

### Characterization of minicollagen expression patterns in different developmental stages

**Figure 4:**
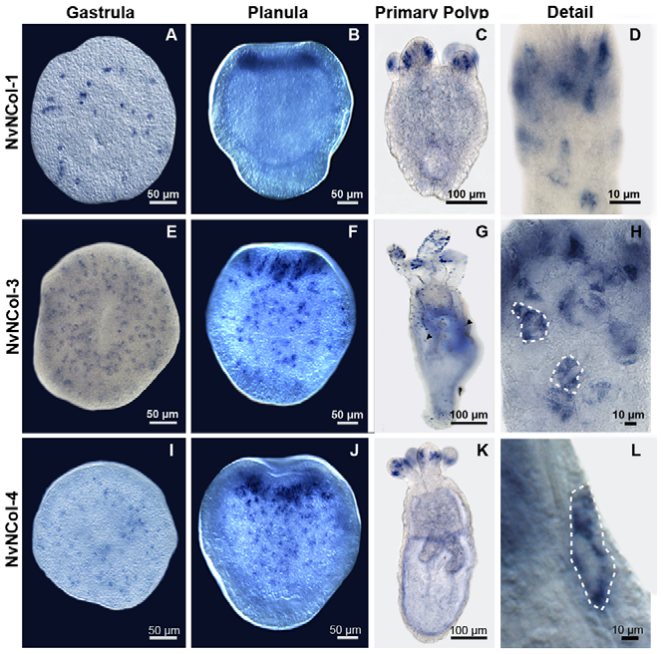
Whole mount *in situ* hybridization for minicollagen genes at different developmental stages of *Nematostella vectensis*. Gene expression patterns for *NvNCol-1* (A–D), *NvNCol-3* (E–H), and *NvNCol-4* (I–L). Arrows in G denote *NvNCol-3* positive signals distributed along the body column. Dotted lines in H and L mark the borders of nematocytes showing positive signals. Scale bars are 100 µm (C, G and K), 50 µm (A, B, E, F, I and J), and 10 µm (D, H and L).

The expression patterns of the isolated minicollagen genes were assayed by *in situ* hybridization (ISH) of whole mounts from different developmental stages (Figure 4). All assayed *Nematostella* minicollagens (NvNCol-1, NvNCol-3 and NvNCol-4) showed a comparable temporal and local expression. Initial signals in the late gastrula stage appeared spot-like and were evenly distributed (Figure 4A, E, I). In planula larvae the expression is significantly increased at the hypostomal area (Figure 4B, F, J). In primary polyps and adult animals minicollagen expression is mainly restricted to the tentacles and the hypostome (Figure 4C, G, K). NvNCol-3 showed additional weaker signals throughout the body column, which were not detectable for the other assayed minicollagens (Figure 4G). Minicollagen expressing cells were cuboidal-shaped epithelial cells and could be detected both, in the ectodermal and entodermal tissue layers (Figure 4D, H, L).

### Localization of nematocyst minicollagens by immunocytochemistry

**Figure 5:**
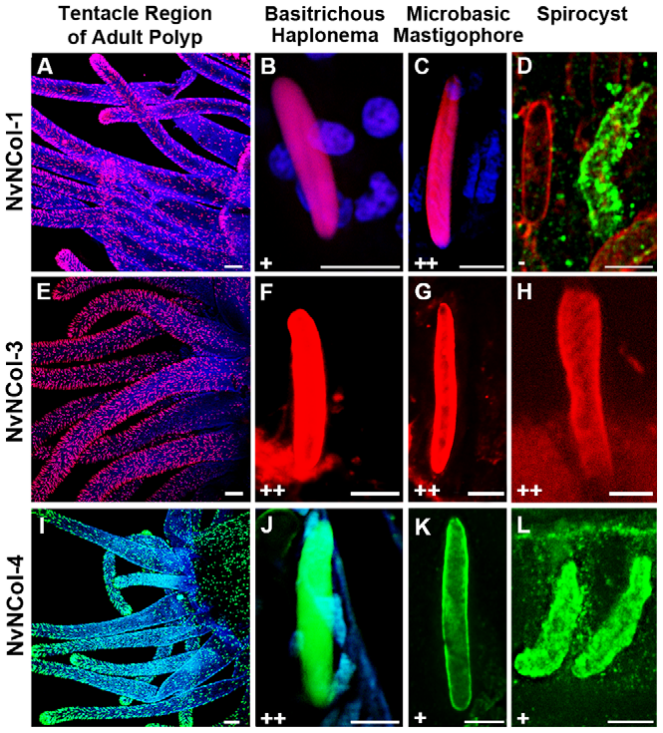
Detection of *Nematostella* minicollagens by immunocytochemistry. Adult polyps were stained with polyclonal minicollagen antibodies and DAPI. A–D. NvNCol-1 staining for different capsule types. D shows a co-staining with NvNCol-4 antibody (green) to demonstrate a lack of NvCol-1 staining in developing spirocysts. E–H. staining for nematocysts using anti-NvNCol-3 antibody. I–L. NvNCol-4 staining of different nematocysts. In A–C and E–H minicollagens were stained with Alexa-568 (red) and in I–L with Alexa-488 (green). + positive signal. ++ strong signal. − no signal. Scale bars are 100 µm (A, E, and I), 10 µm (L), and 5 µm (B, C, D, F, G, H, J, and K).

To analyze the distribution of the different minicollagens on the protein level, we raised polyclonal antibodies against their respective C terminal CRD domains (Figure 3). We have shown that the CRD domains in *Hydra* minicollagens can be used as discriminating antigens for antibody production and do not show cross-reactions with other minicollagen proteins [13]. All minicollagen antibodies used in our study specifically recognized developing nematocysts throughout the animal in adult polyps (Figure 5A, E, I). In contrast to *Hydra*, nematocytes in *Nematostella* do not migrate or form nests of multiple cells, which produce the same nematocyst type. As in the ISH experiment, the tentacles showed the highest density of minicollagen positive cells (Figure S1). Interestingly, the distribution of the different minicollagens among the various capsule types was inhomogeneous. NvNCol-1 could be detected in basitrichous haplonemas and microbasic mastigophores (Figure 5B–C). Spirocysts however, did not show any NvNCol-1 signal. This is demonstrated in Figure 5D by a co-staining with anti-NvNCol-4 antibody (green), which shows a developing spirocyst positive for NvNCol-4 but not for NvNCol-1. NvNCol-3 and NvNCol-4 were detected in all three capsule types with slight variations in their intensity (Figure 5E–L). While the NvNCol-3 antibody yielded strong signals in all capsule types (Figure 5F–H), the anti-NvNCol-4 staining was more pronounced in basitrichous haplonemas (Figure 5J) than in microbasic mastigophores and spirocysts (Figure 5K–L). The anti-NvNCol-1 antibody showed a stronger staining for microbasic mastigophores (Figure 5C) than for basitrichous haplonemas (Figure 5B). All signals detected by the minicollagen antibodies were restricted to the capsule body and did not show any tubule structures. A tubule staining had been expected for NvNCol-1 due to the homology of the cysteine pattern in its truncated C terminal CRD to the one in *Hydra* NCol-15 [13]. In summary, NvNCol-3 appeared to be a basic component of all capsule types, whereas the other minicollagens showed variations in their distribution among different capsule types.

**Figure 6:**
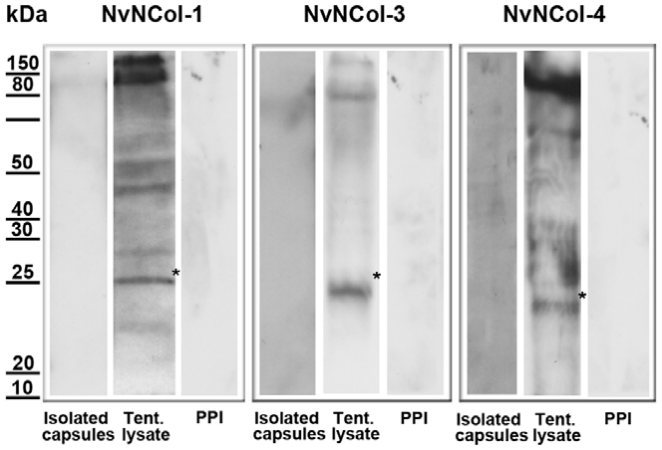
Detection of minicollagens by Western blot analysis. In each lane either isolated capsules or tentacle lysates of one adult head were applied. Preimmune serum (PPI) staining of tentacle lysates was used as negative control for each antibody. The asterisks mark the positions of the putative minicollagen monomer bands.

As in *Hydra*, none of the minicollagen antibodies detected mature capsules, which is probably due to the densely polymerized wall structure. There was, however, a noticeable exception. Mature spirocysts were positive for both anti-NvNCol-3 and NvNCol-4, which might be due to the thinner wall structure of this capsule type (Figure S2). In contrast to *Hydra*, we were not able to detect minicollagen signals in Western Blots of isolated capsules under reducing conditions (Figure 6). *Hydra* nematocysts are extremely sensitive to reducing agents and minicollagens can be readily solubilized from the capsule wall by reduction [11]. *Nematostella* minicollagens were only detectable in tissue lysates containing developing nematocysts, indicating that after maturation minicollagen molecules were not only cross-linked by a disulfide-dependent process but by additional covalent bonds. The calculated molecular masses for the assayed minicollagens are 14.5 kDa for NvNCol-1, 11.2 kDa for NvNCol-3, and 14.7 kDa for NvNCol-4. The detected Western blot bands showed slightly higher apparent molecular masses for the minicollagen monomers (Figure 6), which is probably due to a retarded gel migration of the extended collagen molecules. All minicollagen antibodies detected additional bands at higher molecular masses, which might indicate posttranslational modification and cross-linking. This phenomenon was more pronounced for NvNCol-3 than for the other minicollagens.

## Discussion

The characterization of the *Nematostella* cnidom opens an important evolutionary research perspective as it provides a basis for comparative molecular analyses on a complex organelle system, which probably contributed to the survival of the cnidarian clade since the Cambrian explosion. Furthermore, the reduced cnidocyst repertoire of Anthozoa compared to Medusozoa [16] is believed to reflect a basal state, which could allow insights into the evolutionary origin of nematocysts. Our study clearly defines three cnidocyst types in *Nematostella*, with varying distribution during the different developmental stages and along the body axis. The dominating nematocyst in early development and throughout the body column of all stages is the basitrichous haplonema, while spirocysts are clearly a feature of adult animals and most pronounced in the tentacle region, where they are probably used for prey capture (Figure 2). Spirocysts are often not classified as nematocysts due to their thin wall structure [16]. Our TEM and immunocytochemistry data confirm this notion and in addition demonstrate an apparent lack of a defined opercular structure in this capsule type. On the molecular level, though, our minicollagen stainings clearly demonstrate common protein components between spirocysts and other cnidocysts. Interestingly, spirocysts were the only capsule type, which was not positive for all three minicollagens tested. They lacked a staining for NvNCol-1 (Figure 5D), which is a derived minicollagen with altered CRD patterns. This indicates a simpler molecular architecture of spirocysts, which relies on fewer minicollagen molecules, in particular on NvNCol-3, which is the most conserved member of the protein family. Taken together, spirocysts may represent a primitive form of nematocysts, in which the thickening of the capsule wall and more elaborate structures like opercular flaps and tubule spines have not been realized. The evolutionary process leading to more sophisticated capsule types was apparently accompanied by a diversification of minicollagens. This is emphasized by the fact that the most simplified version of the minicollagen molecule, as in *Hydra* NCol-1 and *Nematostella* NCol-3, is conserved throughout all cnidarians analyzed so far, while minicollagens with more derived domain patterns show a restricted distribution [8].

An intriguing aspect of *Nematostella* minicollagens is the fact that their polymerization appears to involve additional cross-links different from intermolecular cysteine bonds. While minicollagens in *Hydra* can be readily solubilized from mature capsules by treatment with DTT, *Nematostella* minicollagens from mature capsules were resistant to reduction and could only be visualized biochemically in lysates of developing nematocytes. This behavior is reminiscent of fibrillar matrix collagens and might point to a closer relation of *Nematostella* minicollagens to ECM collagens. Such intermolecular cross-links were apparently lost in the more derived nematocysts of medusozoans in which the minicollagen repertoire got significantly expanded.

## Materials and Methods

### *Nematostella* culture

*Nematostella* polyps and embryos were kept in 1/3 seawater (Hand and Uhlinger 1992, Tropic Marine) at 18°C in the dark and fed once or two times a week with *Artemia nauplii*. Induction and gametogenesis were carried out as described before [17]. Oocytes were fertilized *in vitro* to synchronize development.

### Isolation of Nematocysts

Intact, undischarged nematocysts were isolated from *Nematostella* tissue by centrifugation of frozen and thawed hypostomal tissue using isolation solution (50% Percoll, 10% sucrose, 0.003% Triton X-100) at 8200 rpm and 4°C. The isolated capsules were stored in PBS/10% sucrose at −20°C. To increase the yield of spirocysts several runs were done with decreasing Percoll concentration (50–30%).

### Quantitative Nematocyst Analysis

For the quantitative analysis of nematocysts, 50 planula larvae, 50 primary polyps and three adult animals of equal size (sectioned into four parts, and evaluated individually) were dissolved in 2% SDS, spread on a slide and the nematocysts of 6 parallel lanes were counted using brightfield microscopy (Nikon Eclipse 80*i*). The experiment was repeated three times.

#### Microscopy

Fluorescence images, as well as phase contrast and interference contrast images were captured with the Nikon Eclipse 80*i*, confocal images with the Nikon A1R laser-scanning microscope.

### SEM Analysis

Isolated nematocysts were applied to Poly-L-lysine coated cover slides (Sigma-Aldrich Chemie GmbH) and incubated for 10 min in 0.2% glutaraldehyde/2% formaldehyde. After several washes in PBS the procedure was repeated with 2.5% glutaraldehyde/PBS. For dehydration the slides were washed with increasing concentrations of ethanol in PBS before transferring to 100% acetone.

### DNA Sequence Analysis

Commonly used recombinant techniques such as gel electrophoresis, sub-cloning, growth of plasmids, and restriction nuclease digestions were carried out as described by Maniatis et al. (1982). For DNA sequence determination fragments were cloned into a pGEM-T vector (Promega). DNA sequencing was done by Eurofins MWG Operon's sequencing service. Sequence data were analyzed with the sequence analysis software Chromas lite (Technelysium Pty Ltd).

### *In Situ* Hybridization

For fixation, relaxed adult polyps were paralyzed with 3.57% MgCl_2_ in *Nematostella* medium and fixed for 1 h with 4% paraformaldehyde. The polyps were then washed in PBT (PBS and 0.1% Tween20) and incubated for 1 h in MeOH followed by several washes in PBT with decreasing concentrations of MeOH. Polyps were afterwards treated for 5 min with PBT containing 10 µg/ml Proteinase K. Proteinase K digestion was stopped by incubation in 4 mg/ml glycine in PBT for 10 min. Afterwards the adult polyps were incubated for 10 min in 100 mM Triethanolamine (TEA) and 10 min in 100 mM TEA/0.25% acetic anhydride. After two washes in PBT (10 min) the polyps were fixed in 4% paraformaldehyde for 20 min, then washed five times 5 min in PBT and pre-hybridized in a solution containing 50% formamide, 5× SSC, 200 µg/ml yeast-RNA, 0.1% Chaps, 1× Denhardt's, 100 µg/ml heparin, and 0.1% Tween20) for 2 h at 60°C. After adding the heat-denatured, digoxigenin-labeled probe (0.1 ng/µl), samples were hybridized for two days at 60°C followed by several washes in PBT with decreasing concentrations of hybridization solution. The polyps were then incubated for 2 h in blocking solution (1% blocking reagent (Roche) in PBT, 0.02% sodium acid). The hybridized probe was detected using anti-DIG/AP (Roche) at 1/3000 in blocking solution and incubated over night at 4°C. After incubating for 5 min in both NTMT (100 mM NaCl, 100 mM Tris pH 9.5, 50 mM MgCl_2_, 0.1% Tween20) and 1 mM Levamisol/NTMT the staining reaction was performed using NBT/BCIP (Roche ×50 solution) for 45–60 min at 37°C. After staining, the polyps were mounted with PBS/Glycerol and analyzed using the Nikon Eclipse 80*i* microscope with 4×, 20×, 40× and 60× interference contrast optics.

### Immunoblotting

For western blot analysis the capsules were solubilized by heating (95°C, 10 min) in sample buffer (200 mM Tris-HCl pH 6.8, 8% SDS, 0.4% bromphenol blue, 40% glycerin, 1 M β-Mercaptoethanol) The samples were separated by SDS-PAGE using 12% gels, transferred to PVDF membranes and incubated with specific antibodies after blocking for 1 h with 5% skim milk powder in PBS/0.1% Tween 20. The primary antibody was detected using an antibody coupled to horseradish peroxidase (1∶10.000) and the ECL chemoluminescence system (Amersham Biosciences).

### Immunofluorescence

Relaxed animals were paralyzed with 3.57% MgCl_2_ for several minutes and then fixed in Lavdovski's fixative (ethanol∶formaldehyde∶acetic acid∶H_2_O bidest; 50∶10∶4∶36) over night. After several washing steps using PBT (0.1% Triton X-100 in PBS), the polyps were incubated over night at 4°C with NvNCol-1, NvNCol-3 or NvNCol-4 antibodies in PBS/0.1% BSA. Thereafter the polyps were washed several times in PBS and incubated for 2 h with anti-guinea pig (NvNCol-1, NvNCol-3) or anti-rabbit (NvNCol-4) antibody coupled to ALEXA Fluor 568/488 (Molecular Probes) at a 1/400 in PBS/0.1% BSA. The animals were washed again several times in PBS before mounting in PBS/Glycerol. Fluorescence image analysis was performed using the Nikon A1R laser-scanning microscope.

## Supporting Information

Figure S1 **Minicollagen-3 staining of** ***Nematostella*** **whole mounts.** Overview; B. Hypostomal area; C. Body column; D. Foot region. Scale bars are 100 µm.
(TIF)

Figure S2 **Anti-NvNCol-3 staining of mature nematocysts.** A. Mature basitrichous haplonema. B. Mature microbasic mastigophore. C. Mature spirocyst. Scale bars are 5 µm.
(TIF)

Table S1 **Quantitative analysis of capsules at different developmental stages.**
(DOC)
