# Supplementary figures and images for: Morphological and Molecular Analysis of the Nematostella vectensis Cnidom
Source: PLoS One. 2011 Jul 28;6(7):e22725. doi: 10.1371/journal.pone.0022725 (PMC3145756; doi:10.1371/journal.pone.0022725)

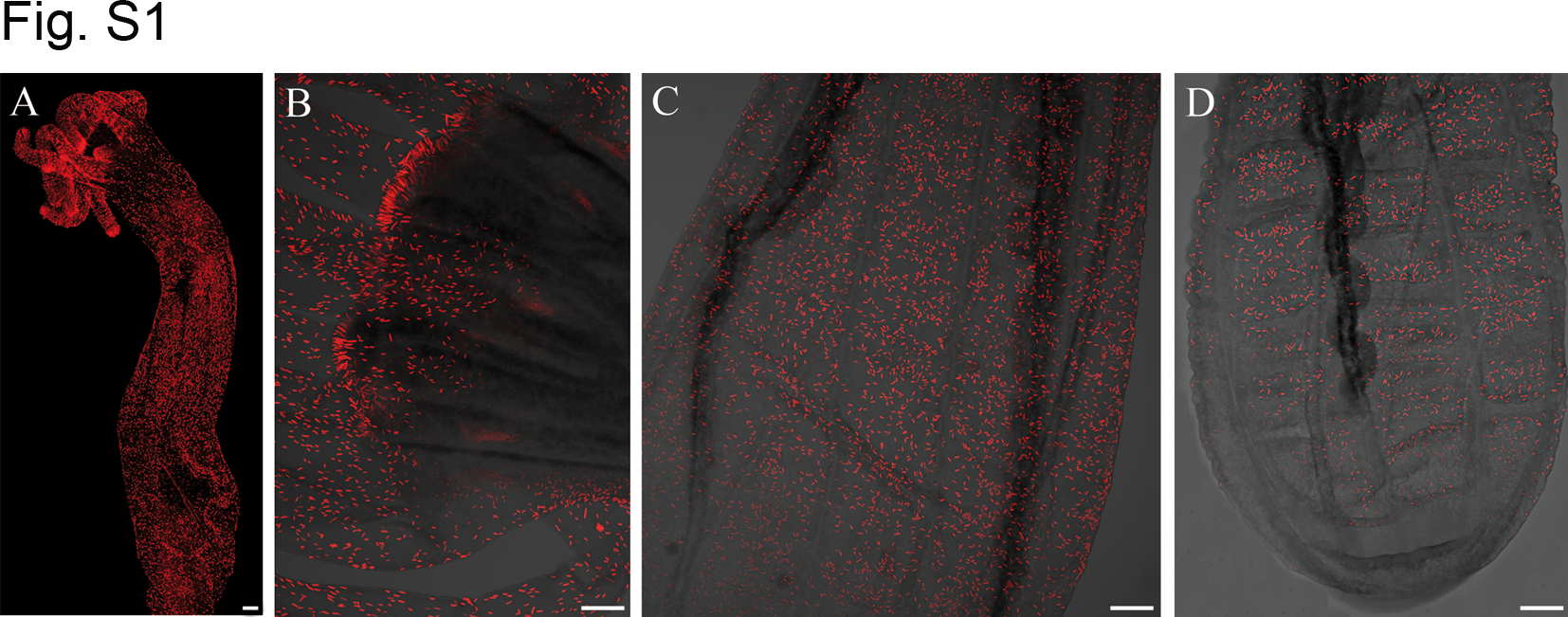

Supplement: Figure S1 — Minicollagen-3 staining of Nematostella whole mounts. Overview; B. Hypostomal area; C. Body column; D. Foot region. Scale bars are 100 µm. (TIF) [file pone.0022725.s001.tif]

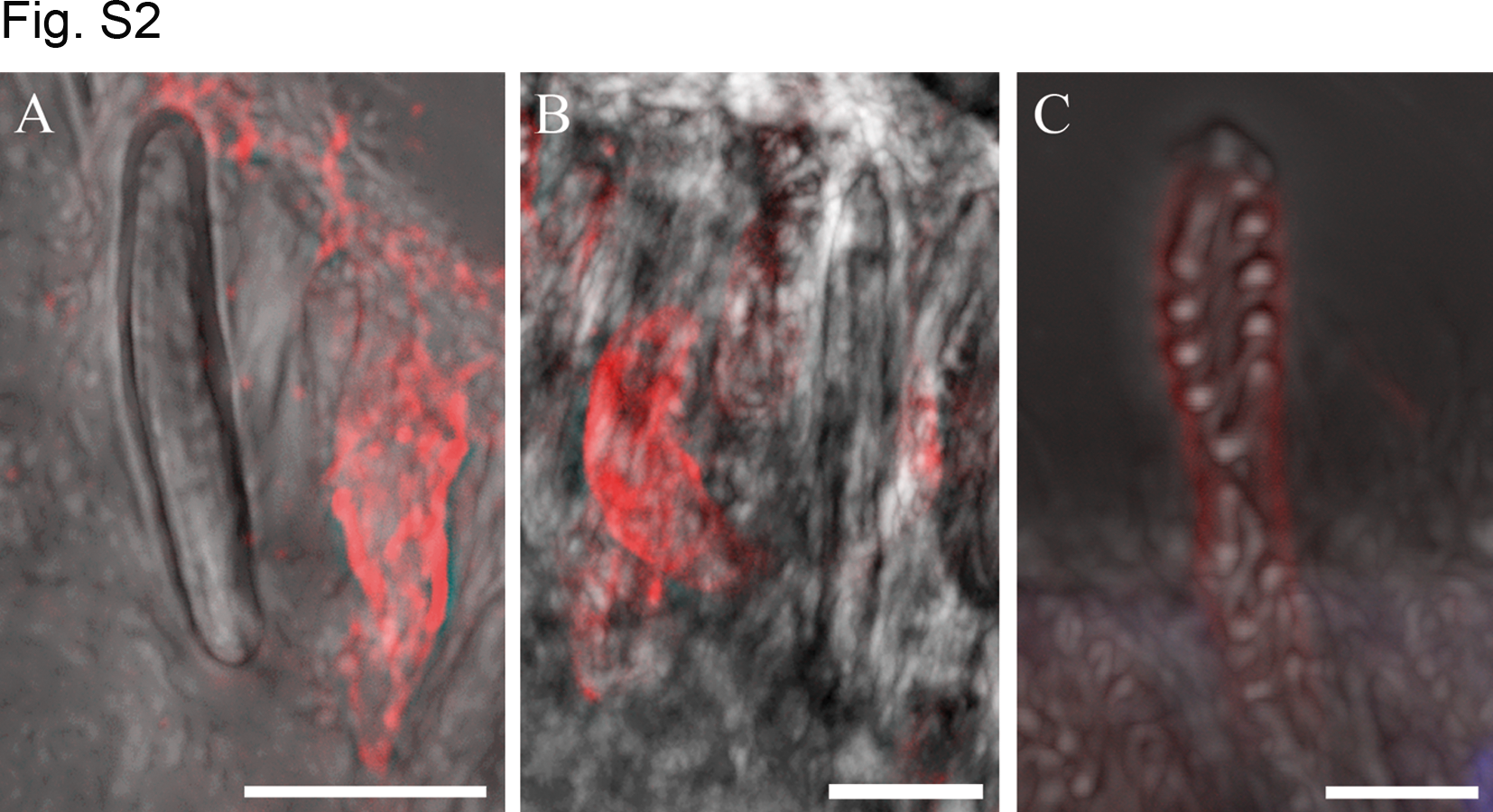

Supplement: Figure S2 — Anti-NvNCol-3 staining of mature nematocysts. A. Mature basitrichous haplonema. B. Mature microbasic mastigophore. C. Mature spirocyst. Scale bars are 5 µm. (TIF) [file pone.0022725.s002.tif]
