# Supplementary material for: Morphological and Molecular Analysis of the Nematostella vectensis Cnidom
Source: PLoS One. 2011 Jul 28;6(7):e22725. doi: 10.1371/journal.pone.0022725 (PMC3145756; doi:10.1371/journal.pone.0022725)
Supplement: Table S1 — Quantitative analysis of capsules at different developmental stages. (DOC) [file pone.0022725.s003.doc]

**Table S1.** Quantitative analysis of capsules at different developmental stages.

|  | Basitrichous Haplonemas | | Microbasic Mastigophores | | Spirocysts | |
| --- | --- | --- | --- | --- | --- | --- |
|  | Total number | % | Total number | % | Total number | % |
| Planula | 838.88 ± 169 | 91.03 ± 4 | 48,91 ± 29 | 5,30 ± 3 | 33.60 ± 10 | 3.65 ± 1 |
| Primary Polyp | 1451.52 ± 363 | 83.38 ± 3 | 253,12 ± 13 | 15,04 ± 3 | 27.63 ± 14 | 1.56 ± 0 |
| Adult Polyp | 411376 ± 87746 | 69.20 ± 1 | 99250,67 ± 28380 | 16,52 ± 3 | 85213.33 ± 23751 | 14.28 ± 3 |
